# A protocol for a systematic review and meta-analysis of tuberculosis care around the time of pregnancy

**DOI:** 10.12688/wellcomeopenres.18072.1

**Published:** 2023-01-10

**Authors:** Camilla Carlsson, Elisabet Lönnermark, Sumona Datta, Carlton A. Evans

**Affiliations:** 1 IFHAD: Innovation For Health And Development, Department of infectious disease, Imperial College London, London, UK; 2 IPSYD: Innovación Por la Salud Y el Desarrollo, Asociación Benéfica PRISMA, Lima, Peru; 3 IFHAD: Innovation For Health And Development, Universidad Peruana Cayetano Heredia, Lima, Peru; 4 Department of Infectious Diseases, Sahlgrenska Academy, University of Gothenburg, Gothenburg, Sweden; 5 Department of Clinical Sciences, Liverpool School of Tropical Medicine, Liverpool, UK; 6 Department of International Health, Johns Hopkins University, Baltimore, USA

**Keywords:** systematic review, meta-analysis, tuberculosis, pregnancy

## Abstract

**Background:** Tuberculosis is estimated to cause 1.5 million deaths annually and is most common during the reproductive years. Despite the fact, we found that tuberculosis screening, prevention or care recommendations for people around the time of pregnancy were absent from some national policy recommendations and varied in others.

**Objectives**: To address the apparent gaps and inconsistencies in policy, we aim to design a systematic review and potential meta-analysis of the original research evidence informing tuberculosis care around the time of pregnancy.

**Methods:** With assistance from librarians at the Biomedical library of the University of Gothenburg, Pubmed, CINAHL and Scopus databases will be searched. Search terms will aim to identify studies generating original research evidence informing care for tuberculosis around the time of pregnancy. Two independent reviewers will screen and select for inclusion the eligible studies. Discrepancies will be resolved with a third reviewer.

We anticipate triaging the eligible publications. Firstly, publications that provide contextual data will be tabulated, summarising their main contributions. Secondly, studies that provide evidence directly guiding patient care and have recently been systematically reviewed and meta-analysed will be tabulated with the recently published conclusions of the syntheses of their data. Thirdly, studies that provide evidence directly guiding patient care, but have not been the subject of recent systematic review and meta-analysis will be our focus and will be considered to be key. The key studies will be subject to quality assessment, data extraction and when possible, meta-analysis.

**Conclusions:** This systematic review and potential meta-analysis aims to guide policy, practice and future research priorities concerning tuberculosis care around the time of pregnancy.

## Introduction

The World Health Organisation (WHO) estimates that tuberculosis (TB) disease causes 1.5 million deaths annually ( WHO, 2022) and that approximately half a million of these deaths are in women ( WHO, 2018). As data on pregnancy are not routinely collected in most TB surveillance programs ( Mathad & Gupta, 2012), the exact number of TB disease cases in pregnancy is poorly characterised. However, TB disease is most common in people of reproductive age and the number of pregnancies affected by TB disease was estimated to be over 200,000 in 2011 ( Sugarman *et al.*, 2014).

Active TB disease during pregnancy is associated with an increased risk of prematurity, low birth weight and perinatal death ( WHO, 2018), and is normally treated with the same regimens as for non-pregnant individuals ( WHO, 2010). However, a recent study has demonstrated that pregnant women have an increased risk of hepatotoxicity and temporary treatment interruptions ( Beck-Friis *et al.*, 2020), possibly indicating a need for closer monitoring of this population as well as for further investigation of the pregnancy-specific safety of TB medications.

To counteract the risks imposed by active TB disease during pregnancy, early TB detection and treatment is important. Most women access antenatal care at least once during pregnancy (UNICEF, 2019), and the integration of TB and antenatal care is recommended by the WHO ( WHO, 2018). Integration could facilitate active TB case findings to increase early detection of the disease, while also helping ensure that the care for concomitant active TB disease and pregnancy is more easily accessible to patients. Another important aspect to include in integrated TB care around the time of pregnancy is family planning, as conception often occurs during TB treatment, and TB medications impair the efficacy of some oral contraceptives. An integrated and holistic approach to TB care around the time of pregnancy may contribute to seeing the pregnant person in the context of their family and extending services further to include family members and household contacts, potentially increasing the impact and reach of interventions within antenatal and TB care.

Early detection and treatment of TB is largely dependent on an efficient screening process. However, there has been recent debate regarding the sensitivity of the often-used method of largely restricting TB diagnostic testing to people with symptoms suggestive of TB. This debate may be particularly important during pregnancy, when symptoms of TB can potentially be masked by or confused with physiologic changes in pregnancy ( Lacourse *et al.*, 2018).

Treatment of active TB disease, regardless of HIV-status, as well as TB preventive therapy (TPT) for latent TB infection in HIV-positive individuals, are generally considered to be necessary even in the event of concurrent pregnancy. By contrast, there is uncertainty concerning the risks versus benefits for TPT during pregnancy for HIV-negative women. It is not yet fully understood if pregnancy and its immune changes increase susceptibility to progression from latent TB infection to active TB disease. However, some studies have demonstrated an increase in TB incidence in the postpartum period ( Gilks *et al.*, 1990; Jonsson *et al.*, 2020; Mathad & Gupta, 2012; Zenner *et al.*, 2012), possibly indicating TB progression during pregnancy and an unmasking of symptoms during the postpartum immune-restitution phase. Whether to recommend TPT during or shortly after pregnancy is still an issue of debate and is a balance between preventing the risks associated with active TB disease during pregnancy and the risks of possible medication side effects. A recent study in HIV-positive women demonstrated greater risks associated with the initiation of isoniazid preventive therapy (IPT) during pregnancy than with initiation postpartum ( Gupta *et al.*, 2019), whereas a systematic review from 2020 concluded that current evidence does not support systematic deferral of IPT until postpartum ( Hamada *et al.*, 2020).

Further adding to the complexities of both active TB disease and latent TB infection around the time of pregnancy are psychological factors intimately associated with pregnancy. These may manifest as an unwillingness to take medications, or to undergo a chest x-ray during pregnancy for fear of harming the foetus. Moreover, they could also cause feelings of guilt in both parents and healthcare personnel if an unfavourable pregnancy event takes place during TB treatment.

The complex interactions between TB and pregnancy outlined above highlight the need for research evidence, and it is therefore regrettable that pregnant women are frequently excluded from research studies.

## Funding

**Figure 1.**
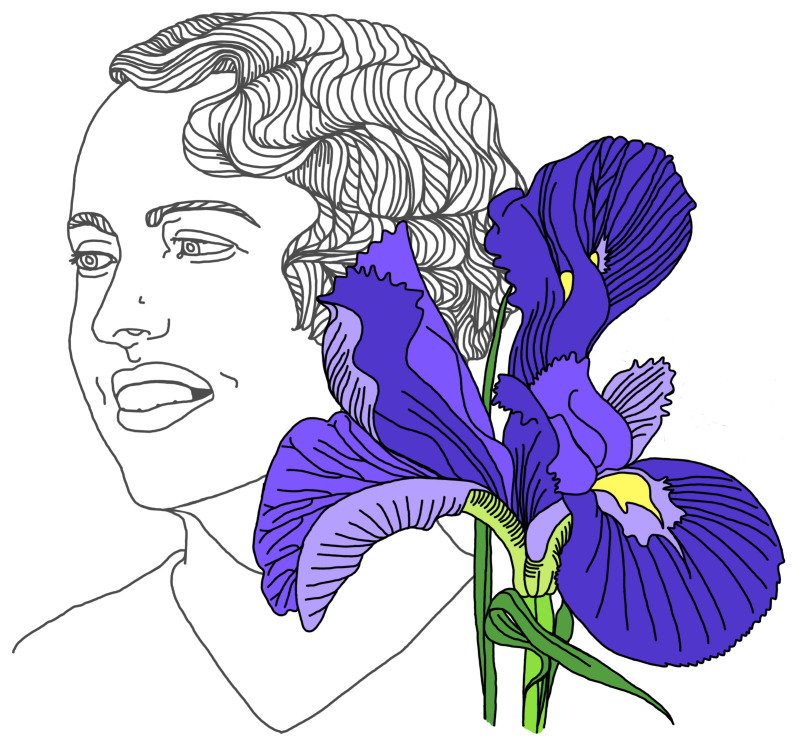
IRIS-stipendiet logo.

This article was supported by Wellcome, Stiftelsen Theodor och Hanne Mannheimers Fond, IRIS-stipendiet ( Figure 1), The United Kingdom Research and Innovation Medical Research Council Skills Development Fellowship, The United Kingdom Research and Innovation Quality-Related Strategic Priorities Fund, The Wellcome fellowship, CONCYTEC/FONDECYT, The Joint Global Health Trials Scheme funding from the Wellcome, UK Foreign, Commonwealth and Development Office, the UK Medical Research Council, and the UK Department of Health and Social Care through the National Institute of Health Research, and IFHAD: Innovation For Health And Development. 

## Survey of current policy documents

The lack of evidence possibly caused by the exclusion of pregnant women from research studies seems to be reflected in international and national guidelines for TB care around the time of pregnancy, as demonstrated by Table 1. The table is the result of a convenience sample where we investigated the international and national guidelines on TB care around the time of pregnancy that were most relevant to the settings where the co-authors work. It illustrates the fact that in many settings where TB screening and/or preventive therapy are recommended for various high-risk groups, pregnancy-specific recommendations are lacking. Furthermore, international and national guidelines that do include pregnancy-related TB screening or preventive therapy recommendations propose strikingly diverse approaches. The apparent gaps and inconsistencies in policy prompted us to undertake a systematic review and meta-analysis to address the following review objective.

**Table 1.** Guidelines for TB screening and treatment during pregnancy.

	TB screening		TB treatment	
	TB infection	TB disease	TB infection	TB disease
**WHO**	No pregnancy-specific recommendations	Partial / selected recommendation *	No pregnancy-specific recommendations	Universal recommendation
**CDC**	No pregnancy-specific recommendations	No pregnancy-specific recommendations	No pregnancy-specific recommendations	Partial / selected recommendation **
**ECDC**	No pregnancy-specific recommendations	No pregnancy-specific recommendations	No pregnancy-specific recommendations	No pregnancy-specific recommendations
**MinSa Peru**	No pregnancy-specific recommendations	No pregnancy-specific recommendations	No pregnancy-specific recommendations	Universal recommendation
**FHM/ILF** **Sweden**	Partial / selected recommendation ***	Partial / selected recommendation ***	Partial / selected recommendation ****	Universal recommendation
**NICE UK**	No pregnancy-specific recommendations	No pregnancy-specific recommendations	No pregnancy-specific recommendations	No pregnancy-specific recommendations

*May be conducted in settings with TB prevalence >100/100 000 **If probability of disease is moderate to high ***If patient is from setting with TB incidence >100/100 000/year or suspected exposure ****During pregnancy if exposed within last 2 years, otherwise deferred to post-partum Note: WHO indicates World Health Organisation, Geneva, Switzerland; CDC indicates Centers for Disease Control, Atlanta, USA; ECDC indicates the European Centres for Disease Control; MinSa indicates Ministerio de Salud (in Spanish, Ministry of Health in English); and NICE indicates National Institute for Health and Care Excellence. “No pregnancy-specific recommendations” indicates that we were unable to identify recommendations specific to pregnancy. “Partial / selected recommendation” indicates that the recommendation is only applicable if a certain condition is met (as specified by the asterisked statement below table). “Universal recommendation” indicates that the guideline included recommendations without requirements for further conditions to be met.

## Review objective

This systematic review and meta-analysis aims to summarise and critically appraise the evidence, informing how best to provide tuberculosis care for people with TB around the time of pregnancy to help guide policy, practice and future research priorities.

## Review question

The review question is intentionally broad in order to capture all the published evidence that can directly guide policy, practice and future research priorities; How should TB care be modified for current or recent pregnancy?

## PICO

### Population

People of any age who are or were recently pregnant, with or without comorbidities such as HIV infection, who have TB or are considered to be at high risk of TB infection or disease.

### Intervention/exposure

Any interventions and/or exposures will be included if they provide evidence informing how best to provide care for people with TB around the time of pregnancy. 

### Comparison

Comparison groups may include people who are not pregnant, or people who do not have TB, but neither comparison group is required by our inclusion criteria.

### Outcome

Outcomes may include but are not restricted to TB prognosis, pregnancy outcome, cost-effectiveness, acceptability, sensitivity and specificity. Outcomes are not required by our eligibility criteria.

## Methods

The systematic review will adhere to the Preferred Reporting Items for Systematic Reviews and Meta-Analyses (PRISMA) checklist.

## Ethics

We do not plan to apply for ethical approval for this systematic review and meta-analysis because no human subjects nor individual participant research data will be involved.

## Inclusion criteria

Original peer reviewed publications in English and/or Spanish, presenting research evidence informing care for TB around the time of pregnancy will be included. There will be no date restrictions on the searches that will include all publications since records began in each database until the date that the searches are last updated, which will be stated in the systematic review publication.

## Exclusion criteria

Publications that do not present original peer reviewed research evidence such as reports, abstracts, editorials, reviews or case reports and studies that cannot inform any aspect of patient care will be excluded. Case series may be eligible if they provide research evidence by including statistical comparison with one or more control groups.

## Publication triage

The included publications will be triaged into three categories. Firstly, publications providing contextual data that is not likely to have an impact on current clinical practices will be tabulated, summarising main findings. Secondly, publications that provide new evidence directly informing clinical practice and patient care and have recently been included in systematic reviews and meta-analysis will be tabulated presenting the recently presented conclusions of the syntheses of their data. Thirdly, publications that provide new evidence directly informing clinical practice and patient care but have not been subject to recent systematic review or meta-analysis will be our focus and will be considered key publications. The key publications will be subject to quality assessment, extraction and summary of key data and if appropriate, meta-analysis.

## Information sources

With assistance from librarians at the Biomedical library of the University of Gothenburg, the following bibliographic database information sources will be searched: Pubmed, CINAHL and Scopus.

## Search strategy for article screening

The databases stated above will be searched with the following search terms:

PubMed:

(Tuberculosis[mesh] OR tuberculosis[tiab] OR tuberculoses[tiab] OR tb[tiab] OR ltb[tiab] OR ltbi[tiab]) AND (Pregnancy[mesh] OR pregnan*[tiab] OR pregnant women[mesh] OR pre-natal[tiab] OR prenatal[tiab] OR post-natal[tiab] OR postnatal[tiab] OR peri-natal[tiab] OR perinatal[tiab] OR postpartum period[mesh] OR post-partum[tiab] OR postpartum[tiab] OR obstetric*[tiab] OR peripartum[tiab] OR peri-partum[tiab]) AND (pregnancy outcome[mesh] OR Outcome[tiab] OR mortality[mesh] OR mortality[tiab] OR Premature Birth[mesh] OR pre-term[tiab] OR preterm[mesh] OR premature[tiab] OR miscarriage*[tiab] OR Abortion, Spontaneous[mesh] OR gestational age[mesh] OR gestational age[tiab] OR stillbirth[mesh] OR stillbirth[tiab] OR stillborn[tiab] still-born[tiab] OR still-birth[tiab] OR congenital[tiab] OR death[mesh] OR death[tiab] OR birth weight[mesh] OR birth weight[tiab] OR birthweight[tiab] OR pregnancy complication*[tiab] OR pregnancy complications[mesh] OR adverse pregnancy outcomes[tiab] OR adverse effect*[tiab] OR adverse event*[tiab])

Limit English, Spanish

The search will then be translated to use in CINAHL and Scopus in addition to Pubmed.

The citations identified will be exported into the systematic review tool Rayyan, where they will be screened by two independent reviewers. Any disagreements will be resolved by discussion with a third independent reviewer. This process will be documented and presented through a flow chart diagram.

## Measures of effect

Effects that have recently been included in any published peer-reviewed systematic review and meta-analysis will be briefly summarised: *e.g.* the safety and efficacy of preventive therapy. Other measures of effect addressing clinically important issues that have not been included in recent meta-analysis will be subjected to quality and bias assessment and, if appropriate, data extraction and meta-analysis.

## Risk of bias (quality) assessment

Bias assessment will be undertaken for the key articles, using a standardised risk of bias assessment tool. The tool will be selected as the most suitable depending on the character of the key studies included in the final selection. We anticipate using a tool designed by the Cochrane group. The Cochrane effective practice and organisation of care (EPOC) risk of bias (RoB) tool may be most appropriate if the key studies on which we focus are all either randomised trials and/or non-randomised trials and/or controlled before-after (CBA) studies and/or interrupted time series (ITS) studies. Further information concerning how we plan to select the most appropriate risk of bias assessment tool is available from the Cochrane group’s guide on how to prepare a risk of bias table for review that include more than one study design, suggested risk of bias criteria for EPOC reviews, and summary assessments of the risk of bias.

## Data extraction

Data will be extracted from the included key articles addressing clinically important information that has not recently been subjected to systematic review or meta-analysis. Data extraction will be done by two to three independent reviewers using a data extraction form in Microsoft Excel (Microsoft Excel for Mac Version 16.66.1). The data extracted will include (but will not necessarily be limited to) study characteristics, methodological characteristics and outcomes.

## Strategy for data synthesis

Data extracted from the key studies will be summarised as follows. Count data will be summarised as proportions with their 95% confidence intervals and represented by bar graphs. Data with an approximately Gaussian distribution will be summarised by means and standard deviations and represented by simple error bar graphs. Strongly skewed data will be summarised by medians and interquartile ranges and may be represented by box plots.

## Meta-analysis

If we find sufficiently similar key studies measuring the same outcome of interest, then a meta-analysis will be performed with a random effects model in order to generate pooled results for presentation in a forest plot. We will assess the heterogeneity of the data with I ^2^ statistics. All data will be analysed using Stata Software version 16.0 (Stata Corporation LLC, College Station, Texas, USA). The meta-analyses will include pooled outcomes of comparable studies calculating their respective weighted means, including weighted confidence intervals.

## Study status

The systematic review is currently in progress, systematically reviewing, assessing eligibility, categorising and extracting data from the eligible literature.

## Dissemination

The work will be published in an international peer reviewed open access journal, ensuring that anyone with internet access can make use of the results.

## Discussion

Our clinical practice has highlighted apparent gaps and variability in TB-related care for people around the time of pregnancy. Consistent with these subjective observations, our summary of selected national and international guidelines identified apparent gaps and inconsistencies in policy. We aim to do a systematic review and meta-analysis of original research evidence informing TB care around the time of pregnancy in order to guide policy, practice and future research priorities.

## Data Availability

All data underlying the results are available as part of the article and no additional source data are required. Harvard database: PRISMA-P checklist for ‘A protocol for a systematic review and meta-analysis of tuberculosis care around the time of pregnancy’. https://doi.org/10.7910/DVN/YD2G3I ( Carlsson & Evans, 2022). Data are available under the terms of the Creative Commons Zero "No rights reserved" data waiver (CC0 1.0 Public domain dedication).
