# Supplementary material for: A protocol for a systematic review and meta-analysis of tuberculosis care around the time of pregnancy
Source: Wellcome Open Res. 2024 Jan 8;8:13. Originally published 2023 Jan 10. [Version 2] doi: 10.12688/wellcomeopenres.18072.2 (PMC10794864; doi:10.12688/wellcomeopenres.18072.2)
Supplement: Peer review history

# Reviewer report: Reviewer response for version 2

**DOI:** 10.21956/wellcomeopenres.23067.r72391

**Published:** 2024-02-16

**Authors:** Dione Benjumea-Bedoya

**Affiliations:** 1 Facultad de Ciencias de la Salud, Corporacion Universitaria Remington (Ringgold ID: 146886), Medellín, Antioquia, Colombia; 2 Universidad de Antioquia, Medellín, Colombia

Most of the reviewer`s comments were addressed, however I consider that the question is still broad and would fit better as a scoping review.

Is the study design appropriate for the research question?

Partly

Is the rationale for, and objectives of, the study clearly described?

Partly

Are sufficient details of the methods provided to allow replication by others?

Partly

Are the datasets clearly presented in a useable and accessible format?

Not applicable

Reviewer Expertise:

Epidemiology of infectious diseases, especially tuberculosis, and evidence-based medicine

I confirm that I have read this submission and believe that I have an appropriate level of expertise to confirm that it is of an acceptable scientific standard.

# Reviewer report: Reviewer response for version 2

**DOI:** 10.21956/wellcomeopenres.23067.r72389

**Published:** 2024-02-01

**Authors:** Alexandra J Zimmer

**Affiliations:** 1 McGill University, Montreal, Québec, Canada

The authors have sufficiently addressed the peer review comments.

Is the study design appropriate for the research question?

Partly

Is the rationale for, and objectives of, the study clearly described?

No

Are sufficient details of the methods provided to allow replication by others?

Partly

Are the datasets clearly presented in a useable and accessible format?

Not applicable

Reviewer Expertise:

Epidemiology, tuberculosis, diagnostics, global health

I confirm that I have read this submission and believe that I have an appropriate level of expertise to confirm that it is of an acceptable scientific standard.

# Reviewer report: Reviewer response for version 2

**DOI:** 10.21956/wellcomeopenres.23067.r72390

**Published:** 2024-01-17

**Authors:** Marian Loveday

**Affiliations:** 1 South African Medical Research Council, University of KwaZulu-Natal, Durban, KwaZulu-Natal, South Africa

The authors of the protocol have addressed the concerns I raised satisfactorily.

Is the study design appropriate for the research question?

Partly

Is the rationale for, and objectives of, the study clearly described?

Partly

Are sufficient details of the methods provided to allow replication by others?

Partly

Are the datasets clearly presented in a useable and accessible format?

Not applicable

Reviewer Expertise:

TB, TB and HIV, DR-TB

I confirm that I have read this submission and believe that I have an appropriate level of expertise to confirm that it is of an acceptable scientific standard.

# Reviewer report: Reviewer response for version 1

**DOI:** 10.21956/wellcomeopenres.20042.r64921

**Published:** 2023-08-23

**Authors:** Alexandra J Zimmer

**Affiliations:** 1 McGill University, Montreal, Québec, Canada

This is an interesting and important research topic. As the authors themselves mention, pregnant persons are often omitted from research studies when it comes to TB. Therefore, this review aims to highlight the knowledge already accumulated and paves the way for future studies, ensuring that this often-overlooked demographic receives appropriate TB care.

 Please consider the following suggestions:

 Abstract

- There's a mention of providing "evidence directly guiding patient care". It might be helpful if you could specify what constitutes "evidence" in this context. Are you referring to a particular intervention, policy, or both?
- The term "potential meta-analysis" is mentioned; however, it's not clear what you might "potentially" meta-analyze. If this hasn't been decided, perhaps consider providing some information about the meta-analysis.

Introduction

- With respect to the number of pregnancies affected by TB disease, consider using a more recent reference. The current reference pertains to data from 2011, which is outdated.
- A citation might be needed for the statement: "and TB medications impair the efficacy of some oral contraceptives."
- The mention of “other recent systematic reviews” appears throughout the protocol. It might be enlightening to discuss these reviews more deeply, focusing on the research questions they addressed and how this review plans to bridge any existing gaps.

Funding

- Figure 1’s direct contribution to the article's content seems limited. Consider omitting it.

Survey of current policy documents

- For clarity, it might be helpful to specify that "co-authors" pertain to the co-authors of this particular manuscript.

Table 1

- It would be advantageous to include a column that indicates the country/region associated with each guideline.
- I notice that the selected countries are those with which the authors have affiliations. Consider the inclusion of guidelines from several high-burden TB countries like India, South Africa, Indonesia, and Kenya might add depth.
- For ease of reading, if space permits, consider placing the recommendation directly within the table cell rather than as a footnote.
- Finally, include references for all guidelines in the table.

Objective / question

- Because the question is broad, the review might be better suited as a scoping review. That said, if other systematic reviews on this subject have already been done, then a scoping review may not be informative. Therefore, I suggest narrowing the research question so that the PICO categories are better defined.

Publication triage

- You cannot exclude articles from your review because other reviews have already covered them. This comes back to my initial question about how this review differs from others that have already been done in this area, and also refining the research question to address research gaps.

Outcomes

- Consider categorizing outcomes as 1) maternal/pregnant person outcomes and 2) infant outcomes.

Measures of effect

- Even with the provided example, the measures being assessed remain a bit ambiguous. Please clarify.

Meta-analysis

- For greater clarity, please define what constitutes a "sufficient" number of studies for conducting a meta-analysis.

Is the study design appropriate for the research question?

Partly

Is the rationale for, and objectives of, the study clearly described?

No

Are sufficient details of the methods provided to allow replication by others?

Partly

Are the datasets clearly presented in a useable and accessible format?

Not applicable

Reviewer Expertise:

Epidemiology, tuberculosis, diagnostics, global health

I confirm that I have read this submission and believe that I have an appropriate level of expertise to confirm that it is of an acceptable scientific standard, however I have significant reservations, as outlined above.

# Response

**Published:** 2024-01-02

**Authors:** Carlton Evans

**Affiliations:** IFHAD: Innovation For Health And Development, Peru

**IMMEDIATELY BELOW ARE ALL THE REVIEWER COMMENTS, EACH FOLLOWED BY OUR RESPONSE** **We thank the reviewer Dr Dione Benjumea-Bedoya for these comments, which we fully quote and reply to below.** **“This is a very interesting systematic review protocol to address the gaps in screening and treatment for latent and active tuberculosis in pregnant and postpartum women. Below are some comments that may help authors to improve their work.** **The IRIS Stipendiet logo is not necessary.”** Thank you for this point. Including this logo in the publication is required by the funder and has been agreed with the journal.

**“Survey of current policy documents: the intention of Table 1 is not clear, especially because the guidelines included are intentionally selected…”** The intention of Table 1 was explained by the statement “The lack of evidence possibly caused by the exclusion of pregnant women from research studies seems to be reflected in international and national guidelines for TB care around the time of pregnancy, as demonstrated by Table 1.”. We have clarified and extended this explanation in the revised version of the manuscript.**** **“…, there are names of institutions, but there is no specific reference for the documents reviewed.”** We have added detailed citation references and weblinks for all these reports in the footnote to Table 1. **“The review question “How should TB care be modified for current or recent pregnancy?” looks different from the objective, and does not follow the PICO question structure.”** Thank you. We have clarified this statement, and we believe that the breadth of our research question is a strength. The detailed PICO question structure immediately follows, in the lines immediately below the research question.

**“The Intervention/exposure is very unspecific.”**

 Thank you we agree and consider the breadth of our inclusiveness of interventions/exposures to be a strength of our approach: “Any interventions and/or exposures will be included if they provide evidence informing how best to provide care for people with TB around the time of pregnancy.”

**“The comparison is not well developed as it should be the comparator of the intervention rather than the population.”**

 We have clarified this statement. **"Results: TB prognosis, pregnancy outcome, cost-effectiveness, acceptability, sensitivity, and specificity should be well described and included as eligibility criteria."** Thank you. We have described these in more detail and also added them to the eligibility inclusion criteria.

**“If this review is so unspecific it should be a scoping review rather than a systematic review and potential meta-analysis.”** We have clarified this important issue by removing mention of a “potential meta-analysis” and clarified that we will perform a systematic review and also a meta-analysis provided that there are three or more studies investigating similar interventions with a similar outcome.

**“Triage of publications: in a systematic review, it is not possible to focus only on publications with new evidence, excluding publications that were already included in previews systematic reviews and meta-analyses, as described in the second triage.”** Thank you, this statement has been deleted from the abstract and manuscript.

**“The information sources did not include grey literature.”** This has been added explicitly to the information sources section.

**“The screening process was not fully described.”** Thank you. We have added more detail to our description of the screening process.    

**We thank** **Marian Loveday for their comments, which we fully quote and reply to below.** **“Thank you for asking me to review this protocol for a systematic review and meta-analysis of tuberculosis care around the time of pregnancy. The authors are undertaking an important and urgently needed task in conducting a systematic review and meta-analysis of TB care around the time of pregnancy and I look forward to reading their paper in the future. The authors have done a great job and the concerns I have raised described below will I hope improve the manuscript. My first concern is the focus of the manuscript. Are you focussing on the care of TB during pregnancy or ‘around the time of pregnancy?’ Although ‘around the time of pregnancy’ is included in the title, the focus of the systematic review and meta-analysis is pregnancy and the research question is ‘How should TB care be modified for current or recent pregnancy?’ The focus of the manuscript needs to be clarified.”** Thank you we regret the previous inconsistency of our wording. We have now standardised our wording to “around the time of pregnancy” in order to accommodate the diverse evidence available. We now clarify that the most relevant research has focused on pregnancy, but that some important research evidence (that should be included in our review) also includes the postnatal period. **“Under the PICO Population section the authors state that their population of interest is ‘People of any age who are or were recently pregnant, with or without comorbidities such as HIV infection, who have TB or are considered to be at high risk of TB infection or disease.’ This needs to be clarified in line with the rest of the manuscript.”** Thank you, we have clarified and made this consistent throughout the manuscript. **“In your methods section you describe that your review and analysis will only include studies that provide evidence directly guiding patient care, but have not been the subject of recent systematic review and meta-analysis will be included. How are you going to identify these studies? Also in your methodology you describe that studies that have recently been included in systematic reviews and meta-analysis will be excluded. Given the limited studies in this field, this may not be many studies and it might be worth considering including studies of maternal TB in the postpartum period.”** Thank you, this statement has been deleted from the abstract and manuscript. **“Minor comments. In the methods section the authors state ‘With the assistance from librarians…’ As far as I am aware, we don’t usually mention the inclusion of the librarians, but just name the databases that will be searched.”** Thank you. The library team have been remarkably helpful and involved in the development of this protocol. We therefore prefer to keep this wording (which has been agreed with the librarians), if this is acceptable to you and the editors. **“This sentence in the abstract is not wrong, but editing will increase its readability: ‘Two independent reviewers will screen and select for inclusion the eligible studies.’ “** Thank you, we have made this improvement. **“Introduction. First paragraph: ‘However, TB disease is most common in people of reproductive age and the number of pregnancies affected by TB disease was estimated to be over 200,000 in 2011.’ I suggest you replace most with more and people with women. Third paragraph: ‘case findings’ should be ‘case finding’ “** Thank you, we have made these improvements. **“Fifth paragraph: ‘…. preventive therapy (TPT) for latent TB infection’ This sentence is not completely correct. TPT is really for the prevention of TB and not the treatment of sub-clinical TB.”** ****Thank you, we have clarified the wording here. **“The section entitled ‘Survey of current policy documents.’ The authors say that ‘The lack of evidence possibly caused by the exclusion of pregnant women from research studies seems to be reflected in international and national guidelines….’ The exclusion of pregnant women from research studies is not the only reason why there is limited evidence, the other reason is the lack of data on TB in pregnant and postpartum women.”** Thank you, we have included this important point. **“Review objective: ‘Aims’ should be ‘aim.’ Outcome:  The authors state that the outcomes may include TB prognosis. Is this supposed to be TB treatment outcomes? If not, this will have to be clarified as to my knowledge, not many studies document ‘TB prognosis.’ “** Thank you, we have corrected these imperfections. **“Study status. From the rest of the text I understood I was reviewing a study protocol, but in the study status it appears the study has already started. This needs to be clarified. “** Thank you, this is complicated by the fact that this protocol manuscript was submitted more than eight months ago, followed by a long delay until peer review comments became available. We have deleted this sentence from this evolving version of the protocol manuscript.  

**We thank** **Alexandra J Zimmer for their comments, which we fully quote and reply to below.** **“This is an interesting and important research topic. As the authors themselves mention, pregnant persons are often omitted from research studies when it comes to TB. Therefore, this review aims to highlight the knowledge already accumulated and paves the way for future studies, ensuring that this often-overlooked demographic receives appropriate TB care. Please consider the following suggestions: Abstract There's a mention of providing "evidence directly guiding patient care". It might be helpful if you could specify what constitutes "evidence" in this context. Are you referring to a particular intervention, policy, or both?”** Thank you we have added a sentence to the abstract specifically clarifying this issue.

**“The term "potential meta-analysis" is mentioned; however, it's not clear what you might "potentially" meta-analyze. If this hasn't been decided, perhaps consider providing some information about the meta-analysis.”** Thank you, this term has been deleted from the abstract and manuscript. M ore information about the meta-analysis has also been added, including clarifying that meta-analysis will be performed if three or more eligible studies are identified. **Introduction. With respect to the number of pregnancies affected by TB disease, consider using a more recent reference. The current reference pertains to data from 2011, which is outdated.”**

 We regret that we are unable to find a more recent reliable estimate and note that recent publications also quote this estimate without any more recent ones. We now comment on this in this section of the manuscript.**** **“A citation might be needed for the statement: "and TB medications impair the efficacy of some oral contraceptives."**

  Thank you, this has been added. **“The mention of “other recent systematic reviews” appears throughout the protocol. It might be enlightening to discuss these reviews more deeply, focusing on the research questions they addressed and how this review plans to bridge any existing gaps.”** Thank you, this statement has been deleted from the abstract and manuscript. **Funding. Figure 1’s direct contribution to the article's content seems limited. Consider omitting it.”** Thank you. Including this logo in the publication is required by the funder and has been agreed with the journal.

**Survey of current policy documents. For clarity, it might be helpful to specify that "co-authors" pertain to the co-authors of this particular manuscript.”** We have made this clarification. **“Table 1. It would be advantageous to include a column that indicates the country/region associated with each guideline.”** Thank you we have added this to the first column of the table.

**“I notice that the selected countries are those with which the authors have affiliations. Consider the inclusion of guidelines from several high-burden TB countries like India, South Africa, Indonesia, and Kenya might add depth.”** Thank you. We agree, but have instead decided to explicitly mention this limitation and we propose this as being potential future research.

**“For ease of reading, if space permits, consider placing the recommendation directly within the table cell rather than as a footnote.”** We agree and have explored various options. We believe that the new layout of Table 1 is much improved and thank the reviewer for this suggestion. **“Finally, include references for all guidelines in the table.”** Thank you we have made this change. **“Objective / question. Because the question is broad, the review might be better suited as a scoping review. That said, if other systematic reviews on this subject have already been done, then a scoping review may not be informative. Therefore, I suggest narrowing the research question so that the PICO categories are better defined.”** Thank you, we have clarified this throughout the manuscript. **“Publication triage. You cannot exclude articles from your review because other reviews have already covered them.”** Thank you, this statement has been deleted from the abstract and manuscript. **“This comes back to my initial question about how this review differs from others that have already been done in this area, and also refining the research question to address research gaps.”** Thank you, we have clarified this throughout the manuscript. **“Outcomes. Consider categorizing outcomes as 1) maternal/pregnant person outcomes and 2) infant outcomes.”** We have made this change. **“Measures of effect. Even with the provided example, the measures being assessed remain a bit ambiguous. Please clarify.”** This paragraph has been re-written, clarified and expanded. **“Meta-analysis. For greater clarity, please define what constitutes a "sufficient" number of studies for conducting a meta-analysis.”** Thank you this is now specified as three eligible studies.   We have also updated the funding statement and references cited.

# Reviewer report: Reviewer response for version 1

**DOI:** 10.21956/wellcomeopenres.20042.r57890

**Published:** 2023-08-23

**Authors:** Marian Loveday

**Affiliations:** 1 South African Medical Research Council, University of KwaZulu-Natal, Durban, KwaZulu-Natal, South Africa

Thank you for asking me to review this protocol for a systematic review and meta-analysis of tuberculosis care around the time of pregnancy. The authors are undertaking an important and urgently needed task in conducting a systematic review and meta-analysis of TB care around the time of pregnancy and I look forward to reading their paper in the future. The authors have done a great job and the concerns I have raised will described below will I hope improve the manuscript.

 My first concern is the focus of the manuscript. Are you focussing on the care of TB during pregnancy or ‘around the time of pregnancy?’ Although ‘around the time of pregnancy’ is included in the title, the focus of the systematic review and meta-analysis is pregnancy and the research question is ‘How should TB care be modified for current or recent pregnancy?’ The focus of the manuscript needs to be clarified.

 Under the PICO Population section the authors state that their population of interest is ‘ *People of any age who are or were recently pregnant, with or without comorbidities such as HIV infection, who have TB or are considered to be at high risk of TB infection or disease.*’ This needs to be clarified in line with the rest of the manuscript.

 In your methods section you describe that your review and analysis will only include studies that provide evidence directly guiding patient care, but have not been the subject of recent systematic review and meta-analysis will be included. How are you going to identify these studies?

 Also in your methodology you describe that studies that have recently been included in systematic reviews and meta-analysis will be excluded. Given the limited studies in this field, this may not be many studies and it might be worth considering including studies of maternal TB in the postpartum period.

**Minor comments**

- In the methods section the authors state ‘ *With the assistance from librarians…*’ As far as I am aware, we don’t usually mention the inclusion of the librarians, but just name the databases that will be searched.
- This sentence in the abstract is not wrong, but editing will increase its readability: ‘ *Two independent reviewers will screen and select for inclusion the eligible studies.*’

**Introduction**

- **First paragraph:** ‘ *However, TB disease is most common in people of reproductive age and the number of pregnancies affected by TB disease was estimated to be over 200,000 in 2011.*’ I suggest you replace most with more and people with women.
- **Third paragraph**: ‘case findings’ should be ‘case finding’
- **Fifth paragraph**: ‘ *….preventive therapy (TPT) for latent TB infection*’ This sentence is not completely correct. TPT is really for the prevention of TB and not the treatment of sub-clinical TB.
- **The section entitled ‘Survey of current policy documents.’**
  The authors say that ‘ *The lack of evidence possibly caused by the exclusion of pregnant women from research studies seems to be reflected in international and national guidelines….*’ The exclusion of pregnant women from research studies is not the only reason why there is limited evidence, the other reason is the lack of data on TB in pregnant and postpartum women.
- **Review objective:** ‘Aims’ should be ‘aim.’
- **Outcome:**  The authors state that the outcomes may include TB prognosis. Is this supposed to be TB treatment outcomes? If not, this will have to be clarified as to my knowledge, not many studies document ‘TB prognosis.’
- **Study status**
  From the rest of the text I understood I was reviewing a study protocol, but in the study status it appears the study has already started. This needs to be clarified.

Is the study design appropriate for the research question?

Partly

Is the rationale for, and objectives of, the study clearly described?

Partly

Are sufficient details of the methods provided to allow replication by others?

Partly

Are the datasets clearly presented in a useable and accessible format?

Not applicable

Reviewer Expertise:

TB, TB and HIV, DR-TB

I confirm that I have read this submission and believe that I have an appropriate level of expertise to confirm that it is of an acceptable scientific standard, however I have significant reservations, as outlined above.

# Reviewer report: Reviewer response for version 1

**DOI:** 10.21956/wellcomeopenres.20042.r59443

**Published:** 2023-08-08

**Authors:** Dione Benjumea-Bedoya

**Affiliations:** 1 Facultad de Ciencias de la Salud, Corporacion Universitaria Remington (Ringgold ID: 146886), Medellín, Antioquia, Colombia; 2 Universidad de Antioquia, Medellín, Colombia

This is a very interesting systematic review protocol to address the gaps in screening and treatment for latent and active tuberculosis in pregnant and postpartum women. Below are some comments that may help authors to improve their work.

 The IRIS Stipendiet logo is not necessary.

 Survey of current policy documents: the intention of Table 1 is not clear, especially because the guidelines included are intentionally selected, there are names of institutions, but there is no specific reference for the documents reviewed.

 The review question “How should TB care be modified for current or recent pregnancy?” looks different from the objective, and does not follow the PICO question structure.

 The Intervention/exposure is very unspecific.

 The comparison is not well developed as it should be the comparator of the intervention rather than the population.

 Results: TB prognosis, pregnancy outcome, cost-effectiveness, acceptability, sensitivity, and specificity should be well described and included as eligibility criteria.

 If this review is so unspecific it should be a scoping review rather than a systematic review and potential meta-analysis.

 Triage of publications: in a systematic review, it is not possible to focus only on publications with new evidence, excluding publications that were already included in previews systematic reviews and meta-analyses, as described in the second triage.

 The information sources did not include grey literature.

 The screening process was not fully described.

Is the study design appropriate for the research question?

Partly

Is the rationale for, and objectives of, the study clearly described?

Partly

Are sufficient details of the methods provided to allow replication by others?

Partly

Are the datasets clearly presented in a useable and accessible format?

Not applicable

Reviewer Expertise:

Epidemiology of infectious diseases, especially tuberculosis, and evidence-based medicine

I confirm that I have read this submission and believe that I have an appropriate level of expertise to confirm that it is of an acceptable scientific standard, however I have significant reservations, as outlined above.
